# Comparative Study Between the Short-Term Effects of Replacement Therapy with Liquid and Tablet Formulations of Levothyroxine on Insulin Resistance Markers in Recently Thyroidectomized Female Patients

**DOI:** 10.3390/metabo15080547

**Published:** 2025-08-13

**Authors:** Francesco Baratta, Federica Moscucci, Raffaella Bocale, Carmine Savoia, Nicholas Cocomello, Ilaria Lospinuso, Evaristo Ettorre, Giovambattista Desideri, Alfredo Pontecorvi

**Affiliations:** 1 Geriatric Unit, Department of Internal Medicine and Medical Specialties, AUO Policlinico Umberto I, 00161 Rome, Italy; federica.moscucci@uniroma1.it (F.M.); ilaria.lospinuso@uniroma1.it (I.L.); 2 Unit of Endocrinology, Department of Translational Medicine and Surgery, Università Cattolica del Sacro Cuore, Fondazione Policlinico “A. Gemelli” IRCCS, 00168 Rome, Italy; raffaella.bocale@policlinicogemelli.it (R.B.); alfredo.pontecorvi@policlinicogemelli.it (A.P.); 3 Clinical and Molecular Medicine Department, Faculty of Medicine and Psychology, Sant’Andrea Hospital, Sapienza University of Rome, 00185 Rome, Italy; carmine.savoia@uniroma1.it; 4 Department of Medical and Cardiovascular Sciences, Sapienza University of Rome, AUO Policlinico Umberto I, 00161 Rome, Italy; nicholas.cocomello@uniroma1.it (N.C.); evaristo.ettorre@uniroma1.it (E.E.); giovambattista.desideri@uniroma1.it (G.D.); 5 Department of Anatomical Sciences, Histological, Legal Medical and Locomotor, Sapienza University of Rome, 00185 Rome, Italy

**Keywords:** levothyroxine, insulin resistance, HOMA-IR, TyG index, thyroidectomy, metabolic syndrome

## Abstract

**Background/Objectives**: Levothyroxine (L-T4) replacement therapy is essential following total thyroidectomy. While liquid L-T4 formulations exhibit superior pharmacokinetic properties compared to tablets, their specific metabolic impact—particularly on insulin resistance—remains unclear. The aim of this study was to compare the short-term effects of liquid versus tablet L-T4 replacement therapy on insulin resistance indices in recently thyroidectomized women and to identify baseline predictors of metabolic response. **Methods**: A post hoc analysis included 130 women randomized to receive either liquid or tablet L-T4 after total thyroidectomy. Metabolic parameters—including the homeostatic model assessment for insulin resistance (HOMA-IR), triglycerides-glucose (TyG) index, and triglycerides-to-HDL cholesterol (TG/HDL-C) ratio—were assessed at baseline and after two months. **Results**: Both L-T4 formulations significantly improved insulin resistance indices over two months. Liquid L-T4 induced a more pronounced reduction in HOMA-IR (treatment effect *p* = 0.022) and fasting insulin levels (treatment effect *p* = 0.017) compared to the tablet formulation. No significant between-group differences were observed for TyG index or TG/HDL-C ratio. Changes in insulin resistance markers were independent of body mass index variations and were predicted by baseline metabolic parameters including insulin, glucose, and lipid levels. **Conclusions**: L-T4 replacement therapy improves insulin resistance markers shortly after thyroidectomy, with the liquid formulation exerting a greater effect on hepatic insulin sensitivity. These findings support the individualized selection of L-T4 formulations to optimize both endocrine and metabolic outcomes post-thyroidectomy.

## 1. Introduction

Levothyroxine (L-T4) replacement therapy is the standard of care after total thyroidectomy to prevent hypothyroidism and maintain physiological thyroid hormone levels [1,2,3]. In clinical practice, L-T4 is commonly administered in tablet form [4]. However, increasing evidence supports the use of liquid formulations [5], particularly in patients with impaired gastrointestinal absorption, altered gastric pH, or those taking multiple medications [6,7,8]. Liquid L-T4 has demonstrated superior pharmacokinetic characteristics, including faster absorption and reduced dependence on gastric acidity, potentially leading to improved biochemical control of thyroid function [6,7,8,9,10].

Thyroid hormones play a crucial role in the regulation of metabolic processes, including lipid and glucose metabolism, energy expenditure, and insulin sensitivity [11,12,13]. Even within the normal reference range, variations in thyroid hormone levels may influence the risk of developing insulin resistance and metabolic syndrome—both recognized precursors of cardiovascular disease [11,14]. Conversely, overt and subclinical hypothyroidism have been associated with increased insulin resistance (IR) and adverse lipid profiles [11,15].

Insulin resistance can be estimated using various surrogate markers. The homeostatic model assessment for insulin resistance (HOMA-IR) reflects hepatic insulin sensitivity [16,17], while newer indices such as the triglycerides-glucose (TyG) index [18,19] and the triglycerides-to-high-density lipoprotein cholesterol (TG/HDL-C) ratio [20] provide information on peripheral insulin resistance and cardiometabolic risk [21]. These indices are simple and cost-effective tools that offer complementary perspectives on insulin sensitivity and have been validated as predictors of type 2 diabetes, atherosclerosis, and cardiovascular disease. Interestingly, previous research demonstrated the beneficial effect of levothyroxine replacement therapy on lipid profile in patients with primary hypothyroidism [22].

Despite the widespread clinical use of different L-T4 formulations, little is known about their specific metabolic effects, particularly in the early post-thyroidectomy period [23]. Investigating the influence of different L-T4 formulations on insulin sensitivity could be of clinical relevance especially in individuals without baseline metabolic disorders, where subtle metabolic changes can be more easily attributed to the intervention. We previously reported a randomized study comparing the effects of liquid and tablet L-T4 formulations on mood states, self-perceived psychological well-being, and thyroid hormone profiles in patients with recent-onset subclinical hypothyroidism after thyroidectomy [9].

The current post hoc analysis expands on that investigation, exploring whether the choice of L-T4 formulation influences early changes in insulin resistance markers in recently thyroidectomized women without malignancy. Our hypothesis was that pharmacokinetic properties of liquid L-T4, likely resulting in more stable thyroid hormone levels and facilitating a more rapid restoration of euthyroidism [9], could more rapidly ameliorate insulin resistance in these patients, eventually representing the best choice in patients with severe metabolic disorders. We used validated metabolic indices—HOMA-IR, TyG index, and TG/HDL-C ratio—to evaluate this effect and to explore baseline predictors of metabolic response, aiming to identify patients who may derive the greatest benefit from a specific formulation.

## 2. Materials and Methods

This is a non-prespecified post hoc analysis of a prospective, randomized, single-center trial with blinded assessment of clinical and laboratory data and blinded statistical analysis, following a PROBE (Prospective Randomized Open Blinded Endpoints) design.

The original study, conducted at the Endocrine Surgery Unit of Università Cattolica del Sacro Cuore (Rome, Italy) investigated the measure of changes in mood states 2 months after initiation of replacement treatment, after surgical thyroidectomy. The secondary outcomes were the measure of changes in self-perceived mental well-being, symptoms/signs of hypothyroidism, thyroid hormone profile and metabolic parameters. The randomization process was performed according to age and sex using SAS version 9.4 [9].

**Figure 1:**
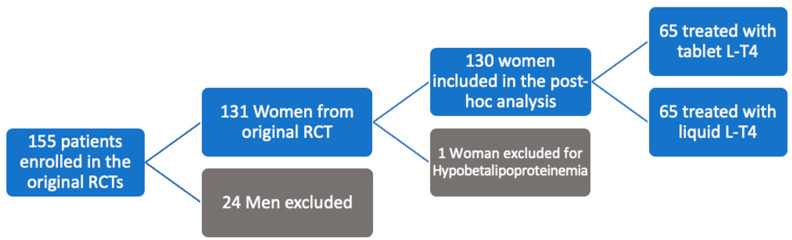
Flow diagram of selection process to identify patients from original randomized clinical trial (RCT) to include in the post hoc-analysis.

The present post hoc analysis was restricted to female participants due to the small number of enrolled males, a consequence of recruitment imbalances and limiting the statistical power for sex-specific analyses. Recruitment imbalances were the probable consequence of the higher incidence of thyroid dysfunction in women [24]. Moreover, one female participant was excluded due to Hypobetalipoproteinemia, a condition that would have confounded the assessment of lipidemic insulin resistance indices. Figure 1 reports the flow diagram of patient selection progress for post hoc analysis.

The original study exclusion criteria included the following: thyroidectomy for malignant disease; clinical conditions requiring increased L-T4 needs (e.g., pregnancy, malabsorption, atrophic gastritis); concomitant use of medications that interfere with L-T4 pharmacokinetics or thyroid function; and the presence of psychiatric disorders or significant comorbidities such as cardiac, cerebral, or renal disease, uncontrolled diabetes mellitus, or chronic alcohol abuse. Participants were randomized within 5–7 days post-thyroidectomy to receive either liquid or tablet L-T4. Dosages were individualized based on body weight (approximately 1.6 mcg/kg/day). Standardized instructions were provided for both formulations, emphasizing ingestion in a fasting state—early in the morning, at least 60 min before consuming any food or drink, including coffee [25,26]. The study was conducted in accordance with the Helsinki Declaration and was approved by the local Ethics Committee of the Policlinico Gemelli—Università Cattolica del Sacro Cuore, ref no 10660/13. Written informed consent was obtained from all participants.

The study was registered under the European Union Clinical Trials Register (EudraCT Number 2013-002139-15).

### 2.1. Post Hoc Aoutcome

The aim of the present post hoc analysis was to investigate the change in insulin resistance indices after two months of L-T4 replacement therapy. The indices evaluated were as follows: HOMA-IR [16,17], TyG index [18,19], and TG/HDL-C ratio [20].

### 2.2. Laboratory Analysis

Fasting blood samples were collected from all participants to measure thyroid hormones, glucose, insulin, and lipid profile. Thyroid-stimulating hormone (TSH), free triiodothyronine (FT3), free thyroxine (FT4), and insulin levels were measured using the COBAS 600 analyzer (Electrochemiluminescence Technology, Roche Diagnostics, Mannheim, Germany). The insulin resistance indices were calculated using the following formulas: HOMA-IR = [fasting insulin (mU/L) × fasting glucose (mmol/L)]/22.5; TyG index = Ln [triglycerides (mg/dL) × glucose (mg/dL)/2]; TG/HDL-C ratio = triglycerides (mg/dL)/HDL cholesterol (mg/dL).

### 2.3. Statistical Analysis

Continuous variables are expressed as mean ± standard deviation (SD). Between-group comparisons were performed using Student’s *t*-test. Treatment and time effects, as well as treatment × time interactions, were evaluated using two-way repeated measures analysis of variance (ANOVA) with the general linear model procedure. Univariate correlations between changes (Δ) in variables were assessed using Spearman’s rank correlation test. Multivariate linear regression analyses using a stepwise approach were conducted to identify baseline predictors of changes in insulin resistance indices. In the evaluation of the treatment’s effect on post hoc outcomes (HOMA-IR, TyG index, and TG/HDL-C ratio), a Bonferroni correction for multiple tests was applied. Considering the 3 different multiple tests and the α = 0.05 of the original RCT, the type I error for post hoc analysis was set to α = 0.01667. The significance of tests for treatment effect was adjusted for multiple tests and indicated as p*.

## 3. Results

In the analysis, 130 women randomized 1:1 for treatment with either liquid or tablet L-T4 were included. The two groups were well balanced for age and body mass index (BMI). No differences were found for metabolic parameters, blood pressure or thyroid hormone profile according to drug formulation (Table 1).

After 2 months of treatment, a difference in thyroid hormone changes was observed between the group assigned to liquid vs. the group assigned to tablet L-T4, confirming the results from the original RCT: TSH (−6.0 ± 5.7 vs. −4.7 ± 5.9, treatment effect *p* = 0.025), FT4 (6.3 ± 4.0 vs. 4.5 ± 4.3, treatment effect *p* = 0.014), and FT3 (1.0 ± 0.6 vs. 0.7 ± 0.9, treatment effect *p* = 0.036).

**Figure 2:**
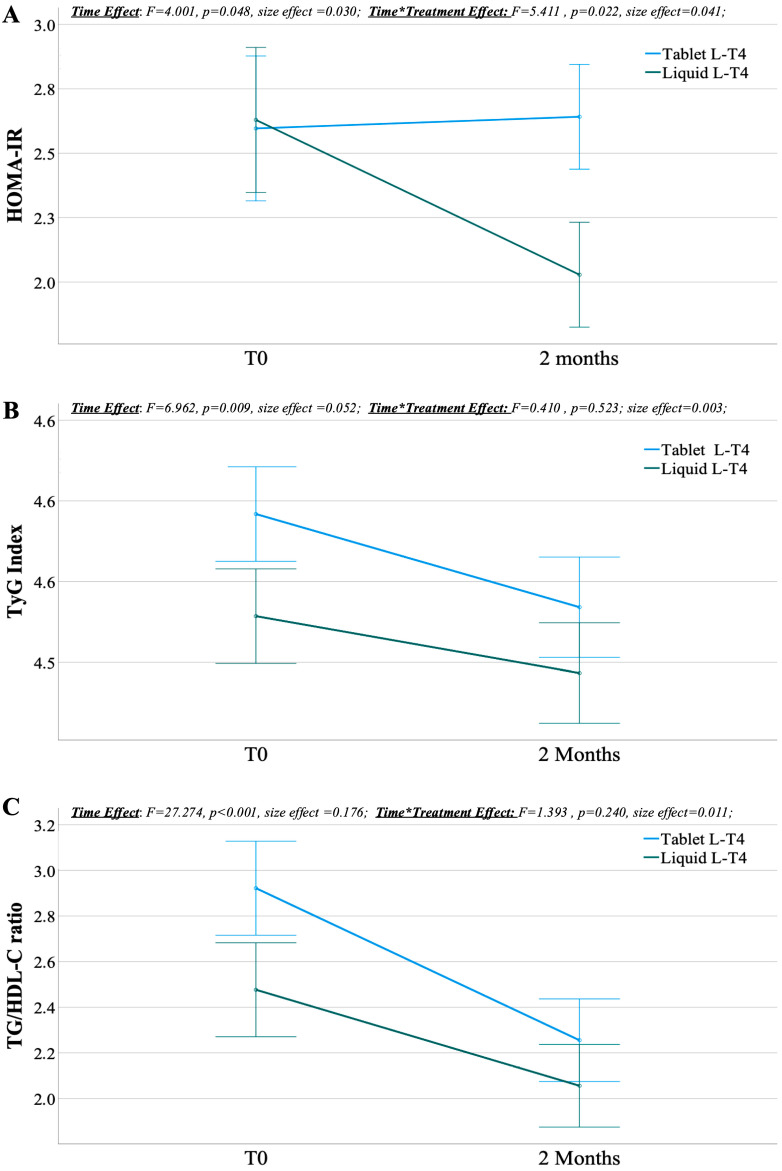
Changes in insulin resistance markers after 2 months under replacement treatment with either liquid or tablet levothyroxine (L-T4) in recently thyroidectomized patients. (**A**) Changes in homeostatic model assessment for insulin resistance (HOMA-IR), (**B**) in triglycerides-glycaemia (TyG) Index, and (**C**) in triglycerides to high-density lipoproteins cholesterol (TG/HDL-C) ratio . Data are presented as mean (symbols) and standard error (bars), α_adjusted_ = 0.017.

Figure 2 reports the time by treatment using a repeated measures ANOVA. Panel A shows a non-significant trend in time by treatment on HOMA-IR after 2 months of L-T4 replacement (F = 5.411, p* = 0.066, size effect = 0.041). While there was no significant HOMA-IR variation with tablet L-T4 (Δ = 0.045, p* = 1.000), the index significantly decreased after treatment with liquid L-T4 (Δ = −0.601, p* = 0.009). Panel B shows no time by treatment effect on the TyG index after L-T4 replacement. Conversely, we found a significant time effect (F = 6.962, p* = 0.027, size effect = 0.052). Considering both formulations together, we observed a significant reduction in the Tyg index (Δ = −0.046, p* = 0.027), but no significance was observed for each treatment alone (tablet L-T4: ΔTyg index = −0.058, p* = 0.066 liquid L-T4; ΔTyG index = −0.035, p* = 0.480). Finally, panel C shows no time by treatment effect on the TG/HDL-C ratio, but shows a significant time effect (F = 27.274, p* < 0.001, size effect = 0.176) with a significant reduction in the TG/HDL-C ratio for both tablet (Δ = −0.667, p* < 0.001) and liquid L-T4 formulations (Δ = −0.421, p* = 0.015).

Table 2 reports the univariate correlations between insulin resistance delta markers and variation in thyroid hormones after 2 months of replacement therapy. None of the insulin resistance changes correlated with Δ BMI. The only correlations observed between Δ thyroid hormones and insulin resistance changes were those between Δ HOMA-IR and Δ TSH (r_s_: −0.206; *p* = 0.019) and between the Δ TG/HDL-C ratio and Δ FT4 (r_S_: −0.297; *p* = 0.016) and Δ FT3 (r_s_: −0.225; *p* = 0.006).

Finally, Table 3 reports the multivariate analyses investigating the baseline predictors of Δ HOMA-IR, the Δ TyG Index, and the Δ TG/HDL-C ratio. Δ HOMA-IR independently correlated with baseline HOMA-IR (Beta: −1.917; *p* < 0.001), insulin (Beta: 1.170; *p* < 0.001), and glucose (Beta: 0.263; *p* = 0.012); Δ TyG independently correlated with baseline TyG (Beta: −0.541; *p* < 0.001), HDL-C (Beta: −0.369; *p* < 0.001), and BMI (Beta: 0.155; *p* = 0.049); the Δ TG/HDL-C ratio independently correlated with the baseline TG/HDL-C ratio (Beta: −0.668; *p* < 0.001), glucose (Beta: 0.232; *p* = 0.002), BMI (Beta: 0.152; *p* = 0.033), HDL-C (Beta: −0.191; *p* = 0.035), and FT3 (Beta: 0.147; *p* = 0.045) (Table 3).

## 4. Discussion

This post hoc analysis of a randomized controlled trial provides new insights into the metabolic effects of L-T4 replacement therapy in recently thyroidectomized women, highlighting a formulation-dependent impact on insulin resistance. Although both tablet and liquid L-T4 effectively improved insulin resistance markers over a two-month period, liquid L-T4 resulted in a greater reduction in HOMA-IR, suggesting a metabolic advantage. The superior improvement in HOMA-IR observed with liquid L-T4 is consistent with prior pharmacokinetic data indicating faster and more consistent absorption of the liquid formulation. These pharmacokinetic properties likely result in more stable thyroid hormone levels, thereby facilitating a more rapid restoration of euthyroidism [6,7,8]. However, the short duration of our follow-up does not allow us to ascertain whether this difference would persist with a longer observation period. In our previous study, we demonstrated that liquid L-T4 led to more pronounced reductions in TSH and greater increases in FT3 and FT4 compared to the tablet formulation [9]. Given the central role of thyroid hormones in modulating hepatic gluconeogenesis and insulin signaling, the enhanced hormonal restoration associated with the liquid formulation may underlie the greater improvement in hepatic insulin sensitivity, as captured by HOMA-IR [27,28]. Interestingly, while both the TyG index and TG/HDL-C ratio, which reflect peripheral insulin sensitivity, significantly improved over time, these changes did not differ by treatment group. The evidence of a short time effect of both formulations on these indexes, reflecting peripheral insulin sensitivity, may demonstrate that both can rapidly obtain beneficial effects on muscles, differently from what observed for the liver. This observation supports the hypothesis that liquid L-T4 may exert its primary metabolic effect on hepatic rather than peripheral insulin resistance [29]. Importantly, changes in insulin resistance markers were not associated with changes in BMI, indicating that the observed metabolic improvements were independent of weight changes. These findings support the notion that L-T4 replacement exerts direct effects on insulin sensitivity beyond those mediated by body composition, despite its well-known effect on body composition [30]. This is consistent with existing literature showing that thyroid hormones regulate insulin receptor expression, glucose transporter activity, and lipid metabolism independently of weight changes [31,32]. Multivariate regression analyses revealed that baseline insulin resistance and metabolic profile significantly predicted treatment response. Specifically, higher baseline HOMA-IR, insulin, and glucose levels were associated with a greater reduction in HOMA-IR. Similarly, baseline lipid parameters and BMI predicted improvements in TyG and TG/HDL-C indices. These findings suggest that metabolic phenotype at baseline may influence the degree of metabolic benefit from L-T4 therapy and highlight the potential value of a personalized approach to replacement therapy selection. Nevertheless, several limitations should be acknowledged. First, the study is a non-prespecified post hoc analysis of a RCT conducted and designed to investigate other aspects than that reported in this study. Second, the study’s short duration limits the long-term metabolic outcomes evaluation. Third, the study population included only Caucasian women under the age of 65, which reduces the generalizability of findings to other populations. Fourth, the use of surrogate indices rather than gold-standard measures such as the hyperinsulinemic-euglycemic clamp may limit the precision of insulin resistance assessment, although the selected indices are validated and commonly used in clinical research. Further studies are needed to investigate the change in surrogate markers of insulin resistance during a longer follow-up period and to test if the same results could be obtained by directly measuring insulin resistance.

## 5. Conclusions

L-T4 replacement therapy significantly improves insulin resistance markers in the early post-thyroidectomy phase, with the liquid formulation demonstrating greater efficacy in reducing hepatic insulin resistance. These findings could support the consideration of the liquid formulation as a preferred option for selected patients and emphasize the importance of individualizing thyroid hormone replacement to achieve not only hormonal but also metabolic optimization.

## Figures and Tables

**Table 1:** Baseline general characteristics of the study participants distinguished according to the treatment assigned.

	Liquid L-T4 (n = 65)	Tablet L-T4 (n = 65)	*p*
Age (years)	51.9 ± 12.6	51.2 ± 13.0	0.774
BMI (kg/m^2^)	26.6 ± 4.9	26.2 ± 5.0	0.909
SBP (mmHg)	124.5 ± 10.0	125.5 ± 9.0	0.588
DBP (mmHg)	76.5 ± 6.3	77.7 ± 7.2	0.309
TC (mmol/L)	4.7 ± 0.8	4.8 ± 1.0	0.384
LDL-C (mmol/L)	2.8 ± 0.7	3.0 ± 0.9	0.339
HDL-C (mmol/L)	1.3 ± 0.3	1.2 ± 0.4	0.303
TG (mmol/L)	1.3 ± 0.5	1.4 ± 0.6	0.174
Glucose (mmol/L)	4.7 ± 1.0	4.9 ± 0.8	0.352
Insulin (mU/L)	11.8 ± 9.0	11.7 ± 7.8	0.932
HOMA-IR	2.6 ± 2.6	2.6 ± 1.9	0.934
TyG Index	4.5 ± 0.2	4.6 ± 0.2	0.129
TG/HDL-C ratio	2.5 ± 1.3	2.9 ± 2.0	0.129
TSH (μU/mL)	8.3 ± 5.6	7.7 ± 4.3	0.491
FT3 (pg/mL)	2.0 ± 0.6	2.1 ± 0.7	0.493
FT4 (pg/mL)	9.2 ± 3.3	9.4 ± 3.6	0.660

BMI: body mass index; SBP: systolic blood pressure; DBP diastolic blood pressure; TC: total cholesterol; LDL-C: low-density lipoprotein cholesterol; HDL-C: high-density lipoprotein cholesterol; TG: triglycerides; HOMA-IR: homeostatic model assessment for insulin resistance; TyG: triglycerides-glycaemia; TSH: thyroid-stimulating hormone; FT3: free tri-iodothyronine; FT4: free thyroxine.

**Table 2:** Relationship between changes (Δ) in thyroid hormones, BMI and insulin resistance indexes.

	Δ HOMA-IR	Δ TyG Index	Δ TG/HDL-C Ratio
Δ BMI (kg/m^2^)	r_s_: 0.016; *p* = 0.855	r_s_: −0.049; *p* = 0.580	r_s_: −0.044; *p* = 0.623
Δ TSH (μU/mL)	r_s_: −0.206; *p* = 0.019	r_s_: 0.084; *p* = 0.344	r_s_: 0.048; *p* = 0.585
Δ FT3 (pg/mL)	r_s_: 0.123; *p* = 0.163	r_s_: −0.065; *p* = 0.460	r_s_: −0.240; *p* = 0.006
Δ FT4 (pg/mL)	r_s_: 0.094; *p* = 0.287	r_s_: −0.089; *p* = 0.315	r_s_: −0.216; *p* = 0.013

BMI: body mass index; HOMA-IR: homeostatic model assessment for insulin resistance; TyG: triglycerides-glycaemia; TG: triglycerides; HDL-C_ high-density lipoprotein cholesterol; TSH: thyroid-stimulating hormone; FT3: free tri-iodothyronine; FT4: free thyroxine; r_s_: Spearman rho.

**Table 3:** Multivariate stepwise linear regression analyses of baseline characteristics associated with insulin resistance index changes (Δ).

**Δ HOMA-IR**					
Model 1	HOMA	Beta: −0.677	B: −0.483	S.E: 0.046	*p* < 0.001
Model 2	HOMA	Beta: −1.301	B: −0.928	S.E: 0.138	*p* < 0.001
	Insulin	Beta: 0.659	B: 0.127	S.E: 0.037	*p* < 0.001
Model 3	HOMA	Beta: −1.917	B: −1.367	S.E: 0.219	*p* < 0.001
	Insulin	Beta: 1.170	B: 0.226	S.E: 0.053	*p* < 0.001
	Glucose	Beta: 0.263	B: 0.463	S.E: 0.182	*p* = 0.012
**Δ TyG Index**					
Model 1	TyG	Beta: −0.355	B: −0.300	S.E: 0.070	*p* < 0.001
Model 2	TyG	Beta: −0.514	B: −0.434	S.E: 0.071	*p* < 0.001
	HDL	Beta: −0.384	B: −0.224	S.E: 0.049	*p* < 0.001
Model 3	TyG	Beta: −0.541	B: −0.456	S.E: 0.071	*p* < 0.001
	HDL-C	Beta: −0.369	B: −0.215	S.E: 0.049	*p* < 0.001
	BMI	Beta: 0.155	B: 0.006	S.E: 0.003	*p* = 0.049
**Δ TG/HDL-C Ratio**					
Model 1	TG/HDL-C ratio	Beta: −0.522	B: −0.372	S.E: 0.054	*p* < 0.001
Model 2	TG/HDL-C ratio	Beta: −0.556	B: −0.396	S.E: 0.051	*p* < 0.001
	Glucose	Beta: 0.296	B: 0.384	S.E: 0.093	*p* < 0.001
Model 3	TG/HDL-C ratio	Beta: −0.577	B: −0.411	S.E: 0.050	*p* < 0.001
	Glucose	Beta: 0.264	B: 0.343	S.E: 0.092	*p* < 0.001
	BMI	Beta: 0.183	B: 0.044	S.E: 0.017	*p* = 0.011
Model 4	TG/HDL-C ratio	Beta: −0.700	B: −0.499	S.E: 0.065	*p* < 0.001
	Glucose	Beta: 0.273	B: 0.355	S.E: 0.091	*p* < 0.001
	BMI	Beta: 0.167	B: 0.040	S.E: 0.017	*p* = 0.020
	HDL-C	Beta: −0.192	B: −0.662	S.E: 0.314	*p* = 0.037
Model 5	TG/HDL-C ratio	Beta: −0.668	B: −0.476	S.E: 0.065	*p* < 0.001
	Glucose	Beta: 0.232	B: 0.301	S.E: 0.094	*p* = 0.002
	BMI	Beta: 0.152	B: 0.037	S.E: 0.017	*p* = 0.033
	HDL-C	Beta: −0.191	B: −0.660	S.E: 0.310	*p* = 0.035
	FT3	Beta: 0.147	B: 0.251	S.E: 0.124	*p* = 0.045

HOMA-IR: homeostatic model assessment for insulin resistance; TyG: triglycerides-glycaemia; HDL-C_ high-density lipoprotein cholesterol; BMI: body mass index; TG: triglycerides; FT3: free tri-iodothyronine.

## Data Availability

The data, analytic methods, and study materials will be made available to other researchers for purposes of reproducing the results or replicating the procedure upon reasonable request by contacting the corresponding author.

## Abbreviations

The following abbreviations are used in this manuscript:

L-T4	Levothyroxine
IR	Insulin Resistance
HOMA	Homeostatic Model Assessment
TYG	Triglycerides-Glucose Index
TG	Triglycerides
HDL-C	High-Density Lipoprotein Cholesterol
TG/HDL-C	Triglyceride-To-High-Density Lipoprotein Cholesterol Ratio
PROBE	Prospective Randomized Open Blinded Endpoints
TSH	Thyroid-Stimulating Hormone
FT3	Free Triiodothyronine
FT4	Free Thyroxine
mg	Milligram
dl	Deciliter
SD	Standard Deviation
ANOVA	Analysis Of Variance
BMI	Body Mass Index
SBD	Systolic Blood Pressure
DBP	Diastolic Blood Pressure
TC	Total Cholesterol
LDL-C	Low-Density Lipoprotein Cholesterol
